# Supplementary material for: Effect of Different Corticosteroid Regimens on the Outcome of Severe COVID-19-Related Acute Respiratory Failure. A Retrospective Analysis
Source: J Clin Med. 2021 Oct 21;10(21):4847. doi: 10.3390/jcm10214847 (PMC8584858; doi:10.3390/jcm10214847)
Supplement: Supplementary file 1 [file jcm-10-04847-s001.zip › jcm-1394228-supplementary.pdf]

Effect of Different Corticosteroid Regimens on the Outcome of Patients with Severe COVID-19-Related Acute Respiratory Failure. A Retrospective Analysis

Paolo Formenti, Michele Umbrello, Stefano Nespoli, Eleonora Pisano, Cecilia Bonino, Stefano Muttini

SUPPLEMENTARY DIGITAL CONTENT

Table S1. Comorbidities, treatment received before ICU admission, blood biochemistry, gas exchange and respiratory physiology at ICU admission, and outcomes of the patients enrolled in the study.

Male sex – n (%)	69 (85.2)
Age (years)	60±10
Height (cm)	172±8
Actual body weight (kg)	82±17
Body mass index (kg/m^2)	27.4±5.2
Predicted body weight (kg)	67±6
SAPS II score	27±7
SOFA score	4 [3; 6]
Frailty score	2 [2; 3]
**Comorbidities – n (%)**	
Hypertension	30 (37.0)
Diabetes	11 (13.6)
Obesity	18 (22.2)
COPD	4 (4.9)
Duration of symptoms before hospitalization (days)	7 [5; 10]
CPAP before ICU admission – n (%)	73 (90.1)
Prone position during CPAP – n (%)	37 (45.6)
Duration of CPAP before intubation (days)	3 [1; 5]
Tracheostomy – n (%)	26 (32.1)
Corticosteroid therapy – n (%)	80 (98.8)
**Type of corticosteroid drug – n (%)**	
Dexamethasone	51 (63.8)
Methylprednisolone	29 (36.2)
Tocilizumab – n (%)	22 (27.2)
**Biochemistry at ICU admission**	
Procalcitonin (μg/L)	0.39 [0.18; 0.89]
C-reactive protein (mg/L)	14.6 [10.0; 22.3]
Fibrinogen (mg/100 mL)	677 [595; 760]
D-dimer (ng/mL)	2121 [764; 4796]
Haemoglobin (g/dL)	11.4±1.8
Platelet count (1/mm^3)	273013±104444
White blood cell (1/mm^3)	9251±4006
Creatinine (mg/dL)	0.85±0.35
Bilirubin (mg/dL)	0.65 [0.43; 1.02]
Interleukin-6 (ng/L)	84 [25; 186]
**Mechanical ventilation and gas exchange at ICU admission**	
Respiratory system compliance (ml/cmH_2O)	49.9±15.2
Airway driving pressure (cmH_2O)	10 [8; 12]
Airway plateau pressure (cmH_2O)	24 [22; 26]
PEEP (cmH_2O)	14 [14; 15]
Tidal volume (mL)	494±53
Tidal volume (mL/kg abw)	7.4±0.8

Respiratory rate (1/min)	19±3
FiO_2 (%)	70.4±14.9
pH	7.37±0.08
PaO_2 (mmHg)	131.8±51.0
$PaCO_2$ (mmHg)	49.4±11.6
PaO_2/FiO_2 (mmHg)	193.3±74.2
**Outcomes**	
Duration of mechanical ventilation (days)	12 [7; 18]
Duration of pressure support ventilation (days)	3 [1; 6]
ICU length of stay (days)	13 [7; 21]
Hospital length of stay (days)	23 [17; 35]
Ventilator-free days (days)	4 [0; 20]
ICU mortality – n (%)	33 (40.7)
Hospital mortality – n (%)	37 (45.7)
Patients who developed VAP – n (%)	40 (49.3)
Number of VAP per patient	2 [1; 2]
Patients who developed bacteremia – n (%)	31 (38.3)
Number of bacteremias per patient	1 [1; 2]
High-dose rescue boluses of methylprednisolone – n (%)	22 (27.2)
Day in which the rescue bolus was administered	6 [4; 9]

SAPS II: Simplified acute physiology score 2nd version; SOFA: Sequential organ failure assessment; COPD: Chronic obstructive pulmonary disease; CPAP: Continuous positive airway pressure; ICU: Intensive care unit; PEEP; Positive end-expiratory pressure; FiO_2: Fraction of inspired oxygen; VAP: ventilator-associated pneumonia

Table S2. Segmented Regression on weekly ICU length of stay data.

Parameter	Estimate	Standard Error	95% CI	p
First period (dexamethasone)				
Slope	3.54	4.68	-5.78; 12.87	0.452
Intercept	21.08	6.79	7.54; 34.61	0.003
Second period (methylprednisolone)				
Slope	2.13	2.20	-2.25; 6.52	0.336
Intercept	14.18	3.19	7.82; 20.54	<0.001
Difference between second and first period				
Slope	-1.41	5.17	-11.72; 8.90	0.786
Intercept	-6.90	7.50	-21.85; 8.05	0.361

Outcome is the length of ICU stay. Linear regression model assessed change in slope and intercept after the change in the corticosteroid drug (methylprednisolone vs. dexamethasone). Time: week since start of study (1–3 dexamethasone and 4-6 methylprednisolone). Intervention: 0 for dexamethasone, 1 for methylprednisolone.

Table S3. Time course of respiratory system compliance, PaO2/FiO2 ratio, SOFA score and the ventilatory ratio at ICU admission, at the time of the bolus, and after 7 and 14 days in patients classified as responders vs. non-responders to the rescue boluses of corticosteroids.

	**Admission (n=22)**	**Bolus (n=22)**	**+7 days (n=19)**	**+14 days (n=19)**	**p (time)**	**p (resp)**	**p (time*resp)**
**Respiratory system compliance (ml/cmH$_2$O)**					**0.0133**	**0.0086**	**0.4440**
Responders	52.1±19.6	50.0±15.6	42.9±10.5	44.8±16.4			
Non responders	49.2±11.7	40.8±10.2	38.8±7.9	31.0±12.2			
**PaO$_2$/FiO$_2$ (mmHg)**					**0.0251**	**<0.0001**	**0.0023**
Responders	179.1±57.9	116.7±18.1	185.0±49.6	209.7±37.5			
Non responders	149.6±62.4	113.2±39.4	108.2±42.3	74.6±19.9			
**Total SOFA**					**0.2444**	**0.0321**	**0.0080**
Responders	4.7±2.3	4.2±1.4	3.5±1.9	3.3±1.3			
Non responders	3.8±1.2	4.3±0.9	4.3±1.3	6.3±2.8			
**Ventilatory ratio**					**0.0004**	**0.0406**	**0.0513**
Responders	1.68±0.26	2.48±0.72	2.21±0.58	2.30±0.55			
Non responders	1.93±0.52	2.16±0.55	2.60±0.48	3.12±1.08			

SOFA: Sequential organ failure assessment score

Table S4. Comparison of baseline characteristics and worst clinical data over the first 10 days of stay in patients who received the rescue boluses of corticosteroids and were classified as responders vs. non-responders.

	Bolus – NR (n=10 – 45.5%)	Bolus – R (n=12 – 54.5%)	p
Male sex – n (%)	8 (88.9)	10 (83.3)	0.7190
Age (years)	58±7	61±8	0.4425
Height (cm)	171±6	172±5	0.8490
Actual body weight (kg)	88±13	81±15	0.3030
Body mass index (kg/m²)	30.0±4.9	27.4±5.2	0.2657
Predicted body weight (kg)	66±5	67±4	0.8612
SAPS II score	25.6±9.1	26.3±4.6	0.8283
SOFA score	4 [3; 4]	5 [3; 7]	0.4479
Duration of symptoms before hospitalization (days)	7 [5; 9]	8 [6; 10]	0.6095
CPAP before ICU admission – n (%)	9 (100)	12 (100)	>0.9999
Prone position during CPAP – n (%)	3 (33.3)	8 (66.7)	0.1301
Duration of CPAP before intubation (hours)	49 [16; 96]	28 [8; 54]	0.4136
Day in which the bolus is administered (days)	6 [2; 9]	6 [5; 9]	0.5176
Tocilizumab – n (%)	5 (55.6)	3 (25)	0.1543
**Biochemistry at ICU admission**			
Procalcitonin (µg/L)	0.33 [0.22; 0.36]	0.51 [0.21; 1.46]	0.2554
C-reactive protein (mg/L)	17.3 [12.9; 24.9]	20.0 [13.4; 22.9]	0.9716
Fibrinogen (mg/100 mL)	760 [680; 786]	677 [612; 786]	0.4318
D-dimer (ng/mL)	4128 [1670; 4631]	978 [581; 8194]	0.7573
Bilirubin (mg/dL)	0.56 [0.49; 1.07]	0.55 [0.38; 0.75]	0.6185
Interleukin-6 (ng/L)	135 [38; 359]	237 [148; 164]	0.5677
**Worst respiratory mechanics and gas exchange before the bolus**			
Respiratory system compliance (ml/cmH₂O)	40.8±10.2	50.0±15.6	0.1403
Airway driving pressure (cmH₂O)	13 [11; 15]	13 [11; 17]	0.8853
Airway plateau pressure (cmH₂O)	28 [23; 28]	26 [22; 30]	0.8865
PEEP (cmH₂O)	13 [12; 15]	12 [10; 13]	0.0910
Tidal volume (mL)	511±43	574±96	0.0842
Respiratory rate (1/min)	21±2	20±3	0.2170
FiO_2 (%)	78.8±11.4	65.4±14.7	0.0343
pH	7.43±0.06	7.38±0.07	0.1203
PaO_2 (mmHg)	87.6±26.5	75.2±15.9	0.1931
$PaCO_2$ (mmHg)	49.6±8.0	54.1±11.7	0.3336
PaO_2/FiO_2 (mmHg)	113.2±39.4	116.7±18.1	0.7909
SOFA score (points)	4.6±1.0	5.4±1.6	0.1673

SAPS II: Simplified acute physiology score 2nd version; SOFA: Sequential organ failure assessment; COPD: Chronic obstructive pulmonary disease; CPAP: Continuous positive airway pressure; ICU: Intensive care unit; PEEP; Positive end-expiratory pressure; FiO_2: Fraction of inspired oxygen
